# Structural and functional diversity of free-living microorganisms in reef surface, Kra island, Thailand

**DOI:** 10.1186/1471-2164-15-607

**Published:** 2014-07-18

**Authors:** Naraporn Somboonna, Alisa Wilantho, Anunchai Assawamakin, Somchai Monanunsap, Duangjai Sangsrakru, Sithichoke Tangphatsornruang, Sissades Tongsima

**Affiliations:** 1 Department of Microbiology, Faculty of Science, Chulalongkorn University, Bangkok 10330, Thailand; 2 Genome Institute, National Center for Genetic Engineering and Biotechnology, Khlong Nueng, Khlong Luang, Pathum thani 12120, Thailand; 3 Department of Pharmacology, Faculty of Pharmacy, Mahidol University, Bangkok 10400, Thailand; 4 Department of Marine and Coastal Resources, Ministry of Natural Resources and Environment, Bangkok 10400, Thailand

**Keywords:** Biodiversity, Prokaryote, Eukaryote, 16S ribosomal RNA, 18S ribosomal RNA, Reef surface, Pyrosequencing

## Abstract

**Background:**

Coral reefs worldwide are being harmed through anthropogenic activities. Some coral reefs in Thailand remain well-preserved, including the shallow coral reefs along Kra island, Nakhon Si Thammarat province. Interestingly, the microbial community in this environment remains unknown. The present study identified biodiversity of prokaryotes and eukaryotes of 0.22-30 μm in sizes and their metabolic potentials in this coral reef surface in summer and winter seasons, using 16S and 18S rRNA genes pyrosequencing.

**Results:**

The marine microbial profiles in summer and winter seasons comprised mainly of bacteria, in phylum, particular the Proteobacteria. Yet, different bacterial and eukaryotic structures existed between summer and winter seasons, supported by low Lennon and Yue & Clayton theta similarity indices (8.48-10.43% for 16S rRNA, 0.32-7.81% for 18S rRNA ). The topmost prokaryotic phylum for the summer was Proteobacteria (99.68%), while for the winter Proteobacteria (62.49%) and Bacteroidetes (35.88%) were the most prevalent. Uncultured bacteria in phyla Cyanobacteria, Planctomycetes, SAR406 and SBR1093 were absent in the summer. For eukaryotic profiles, species belonging to animals predominated in the summer, correlating with high animal activities in the summer, whereas dormancy and sporulation predominated in the winter. For the winter, eukaryotic plant species predominated and several diverse species were detected. Moreover, comparison of our prokaryotic databases in summer and winter of Kra reef surface against worldwide marine culture-independent prokaryotic databases indicated our databases to most resemblance those of coastal Sichang island, Chonburi province, Thailand, and the 3 tropical GOS sites close to Galapagos island (GS039, GS040 and GS045), in orderly.

**Conclusions:**

The study investigated and obtained culture-independent databases for marine prokaryotes and eukaryotes in summer and winter seasons of Kra reef surface. The data helped understand seasonal dynamics of microbial structures and metabolic potentials of this tropical ecosystem, supporting the knowledge of the world marine microbial biodiversity.

## Background

Previous studies reported different species and species distribution patterns in different coastal and open ocean environments, climates, distances from seashores, and sea depths [1-4]. Dinsdale and colleagues [5] described different prokaryotic and viral communities across 4 coral atolls of the Northern Line Islands, influenced by oceanographic conditions and human activities associated with land-use and fishing. Human activities are considered a major factor driving microbial structure changes [4,6-8]. Somboonna et al. [4] reported that two opposite coastal niches of the non-vast Sichang island with different degrees of manmade pollutions contained diverse microbial structures. Furthermore, seasonal variation affects the microbial diversity. Studies described the repeatable seasonal dynamic of the microbial structures in temperate coastal water of N50.2518 W4.2089 with peak biodiversity in the winter season [9,10]. Yet, the seasonal dynamic of the microbial structure of the tropical coral reef remains unrevealed.

Coral reefs of tropical waters between 30°N and 30°S are generally around shallow depth. This shallow-water reef zone, known as reef surface, is affected by the surge of water tides that further enhances the biodiversity. Although coral reefs cover less than 1% of the Earth’s surface, they are home to approximately 25% of marine fish species [11-14]. Corals, coral animals and microbiota have intricate relationship. For instances, coral animals and microbiota could protect the corals from water temperature rise, pollution, and also from specific pathogens by filling entry niches and/or producing antibiotics [5,15,16]. Changing of microbial associates could help the coral animals adapt to altering coral niches [15,17]. In contrast, coral bleaching causes change to the microbial community balance [18]. Thus, it is of importance to understand these dramatically fruitful marine microbial communities.

Currently, culture-independent method has been in widespread use to obtain microbial databases for marine and other various environmental resources [1,11,13]. This derived the global ocean sampling exploration (GOS) project that was launched in 2003 by JC Venter to gain understanding of microbial diversity for entire marine environments, including coastal water, open ocean, seafloor and seawater at different depths [1,3,4,19-21]. While no study for culture-independent microbial diversity of any coral reef environment in Thai maritime has been established, this study represents the first to use 16S and 18S rRNA pyrosequencing with metagenomic DNA to identify the summer and winter microbial structures and their metabolic potentials representing Thailand’s tropical reef surface of Kra island, helping to understand our global marine biodiversity.

**Figure 1:**
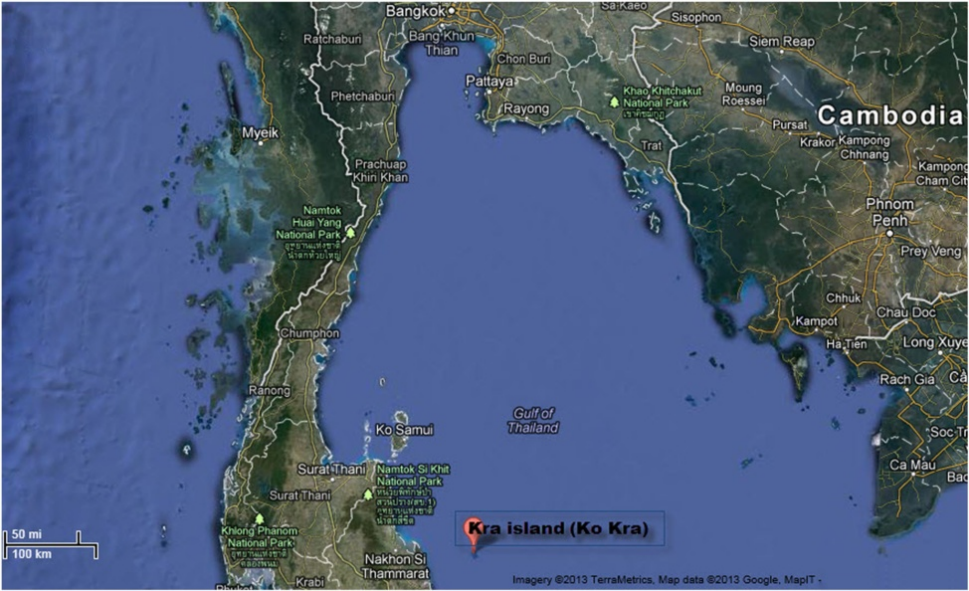
**Satellite map of Kra island.** The map was from Google Satellite Map, retrieved on 7 May 2013, from http://maps.google.com/. Red represents Kra island.

Being situated just above the equator makes Thailand a tropical climate with great biodiversity of microbes and organisms. Kra island, or Ko Kra, is located in the Gulf of Thailand at N8.39817 E100.73283, about 437 miles (700 km) south of Bangkok and 34 miles (54 km) east of Nakhon Si Thammarat province of Thailand (Figure 1). The coral reefs around Kra island are still in well-preserved condition due to the minimal anthropogenic impact, meanwhile the world’s coral reefs have been dramatically degraded in the past few decades [14]. Kra island is uninhabited as it has a small size of less than 0.1 square miles and is remote from the mainland.

## Results

### General characteristics of Kra reef surface

Three independent water samples representing Kra island reef surface were collected at N8.40116 E100.73232, N8.39768 E100.73643 and N8.36135 E100.73524, during midday in summer (May 2011) and winter (January 2012) seasons of Thailand. All collected water samples were clean and had no abnormal smell. On-site physical and chemical property measurements of the collected water samples expressed similar pH, salinity and temperature between the two seasons, while dissolved oxygen was higher and suspension solids were fewer in the winter (Table 1).

**Table 1:** General water properties

**Seasons**	**pH**	**Salinity (ppt)**	**Temperature (ºC)**	**Dissolved oxygen (mg/L)**	**Suspension solids (mg/L)**
Summer	7.5	31.7	30.2	6.3	13.1
Winter	7.5	32.0	29.1	7.0	11.9

Following a two-step water filtration system to capture marine microbes or particles of 0.22-30 microns in diameters [4,22] and total nucleic acids extraction, the average metagenomic DNA retrieved for the summer and the winter samples were 0.22 and 0.19 nanogram per millilitre of seawater, respectively.

### Species compositions at domain and taxon levels

**Figure 2:**
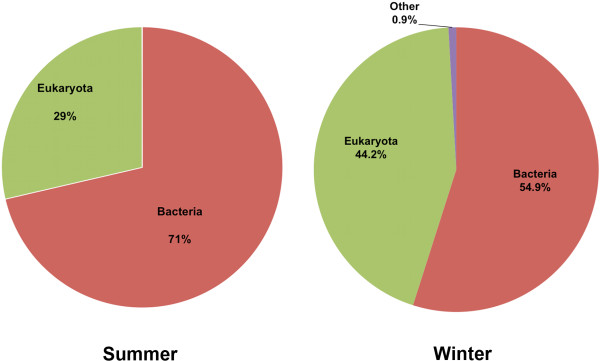
**16S and 18S rRNA genes overview for domain compositions.** “Other” denotes species not belong to Bacteria and Eukarya domains by mg-RAST.

**Figure 3:**
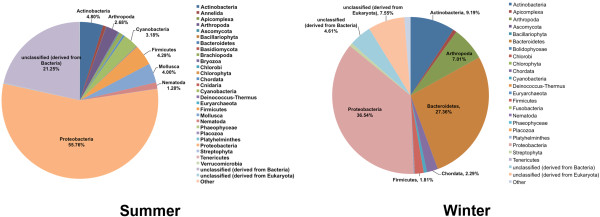
**16S and 18S rRNA genes overview for taxonomic compositions.** Taxons with minor abundance were shown in corresponding colors but were not name listed in the circular diagram.

Libraries of summer and winter 16S and 18S rRNA genes sequences were successfully constructed and sequenced: 66,600 total reads were classified into 14,189 reads for summer 16S rRNAs, 42,659 for winter 16S rRNAs, 1,709 for summer 18S rRNAs, and 8,043 for winter 18S rRNAs. The average number of reliable reads (having read length of greater than or equal to 50 nucleotides) was 97.43% and the average read length was 186 nucleotides. 96.23% of the reliable reads could be identified by BLASTN against NCBI [23], RDP [1,24] and Greengenes [25] for 16S rRNA genes; and NCBI [23], EMBL [26,27] and SILVA [28] for 18S rRNA genes. Similarly, analysis by mg-RAST (metagenomics - Rapid Annotation using Subsystems Technology) [29,30] revealed high numbers of reliable reads: 95.9% for summer and 99.2% for winter.Comparing between Bacteria and Eukarya domains using mg-RAST, Bacteria domain was more present than Eukarya in both seasons, although the great proportion of Eukaryota was found in the winter (Figure 2). Different taxonomic compositions between the seasons were demonstrated: summer mainly comprised taxons Proteobacteria, unclassified (derived from bacteria), Actinobacteria, Firmicutes, Mullusca, Cyanobacteria, Arthopoda, Nematoda, Brachiopoda, and Annelida, in orderly. The winter mainly comprised of taxons Proteobacteria, Bacteroidetes, Actinobacteria, unclassified (derived from eukaryotes), Arthropoda, unclassified (derived from bacteria), Chordata, Firmicutes, and Apicomplexa (Figure 3).

It is important to note that the varied pyrosequencing depths between summer and winter 16S and 18S rRNA datasets were normalized by random selection of equal number of reads from both summer and winter seasons for comparative analysis of microbial communities. 10,000 random reads of each 16S and 1,600 reads of each 18S rRNA datasets were examined for distributions by taxons (Additional file 1: Figure S1) and phyla (Additional file 2: Table S1). These distribution patterns were similar to those computed using the entire datasets (Figure 3 and Table 2). The normalization analyses were independently performed twice, and the results remained the same. Additionally, rarefaction cures of the number of phyla on the Y-axis against the number of random reads on the X-axis were constructed for 16S and 18S rRNA datasets, and the plateau curves showing relatively no new phyla at these sequencing depths were observed for the 16S and 18S rRNA data.

**Table 2:** Percent compositions of free-living prokaryotic (A) and eukaryotic (B) phyla in summer and winter seasons

**A**		
**Phyla**	**Summer (%)**	**Winter (%)**
Proteobacteria	99.681	62.950
Actinobacteria	0.082	0.739
Bacteroidetes	0.208	36.146
Firmicutes	0.030	0.143
SAR406	-	0.005
Planctomycetes	-	0.005
Cyanobacteria	-	0.010
SBR1093	-	0.002
**B**		
**Phyla (Kingdom)**	**Summer (%)**	**Winter (%)**
Ascomycota (Fungi)	0.924	1.534
Basidiomycota (Fungi)	0.231	0.039
Glomeromycota (Fungi)	0.115	-
Neocallimastigomycota (Fungi)	-	0.052
Zygomycota (Fungi)	-	0.039
Apicomplexa (Protist)	-	1.403
Ciliophora (Protist)	-	1.062
Dinophyta (Protist)	10.855	0.354
Mycetozoa (Protist)	-	0.184
Bacillariophyta (Plant)	1.617	72.473
Chlorophyta (Plant)	0.231	0.197
Cryptophyta (Plant)	0.346	0.118
Eustigmatophyceae (Plant)	-	0.013
Haptophyta (Plant)	-	0.118
Phaeophyceae (Plant)	-	0.354
Pinguiophyceae (Plant)	-	0.262
Stramenopiles (Plant)	-	14.825
Streptophyta (Plant)	0.115	0.105
Xanthophyceae (Plant)	0.115	0.669
Annelida (Animal)	15.589	0.184
Arthropoda (Animal)	6.236	0.079
Brachiopoda (Animal)	37.067	0.013
Chordata (Animal)	1.039	0.734
Cnidaria (Animal)	-	0.459
Gastrotricha (Animal)	0.231	-
Mollusca (Animal)	23.672	-
Placozoa (Animal)	-	4.706
Platyhelminthes (Animal)	1.039	0.013
Porifera (Animal)	-	0.013
Rotifera (Animal)	0.557	-

Each identified read was classified in its corresponding phylum. The proportional percentage of each phylum was calculated by dividing the number of the identified reads in a phylum with the total number of the identified reads.

### Prokaryotic diversity of summer and winter Kra reef surface

As an additional analysis to the mg-RAST analysis for domain and taxon compositions, the 16S rRNA sequence profiles of summer and winter Kra prokaryotes were species annotated by BLASTN [31] with E-value threshold ≤10^−5^ against NCBI non-redundant [23], RDP [24] and Greengenes [25] databases. With added databases, most unclassified (derived from Bacteria) by mg-RAST analysis became annotated. The summer profile demonstrated an overwhelming proportion of Proteobacteria (99.68%), whereas the winter profile constituted the higher phyla diversity: Proteobacteria (62.49%), Bacteroidetes (35.88%), and the greater proportion of Actinobacteria (8.95-fold) and Firmicutes (4.73-fold) than those in the summer (Table 2A). Prokaryotic phyla, including Cyanobacteria, SAR406, Planctomycetes and SBR1093, were only detected in the winter (Table 2A). SAR406 (from Greengenes database) represents uncultured bacteria that were previously discovered in Atlantic and Pacific oceans. Its full-length 16S rRNA phylogeny was not clustered with any bacterial phyla except was a distant relative to genera *Fibrobacter* (the only genus of phylum Fibrobacteres, capable of degrading plant fiber) and *Chlorobium* (green sulfur bacteria of phylum Chlorobi) [32]. SBR1093 (a relative of green sulfur bacteria *Chlorobium* in Chlorobi; from Greengenes database) represents uncultured bacteria which previous research found in wastewater treatment plants with biological nutrient removal [33].

**Figure 4:**
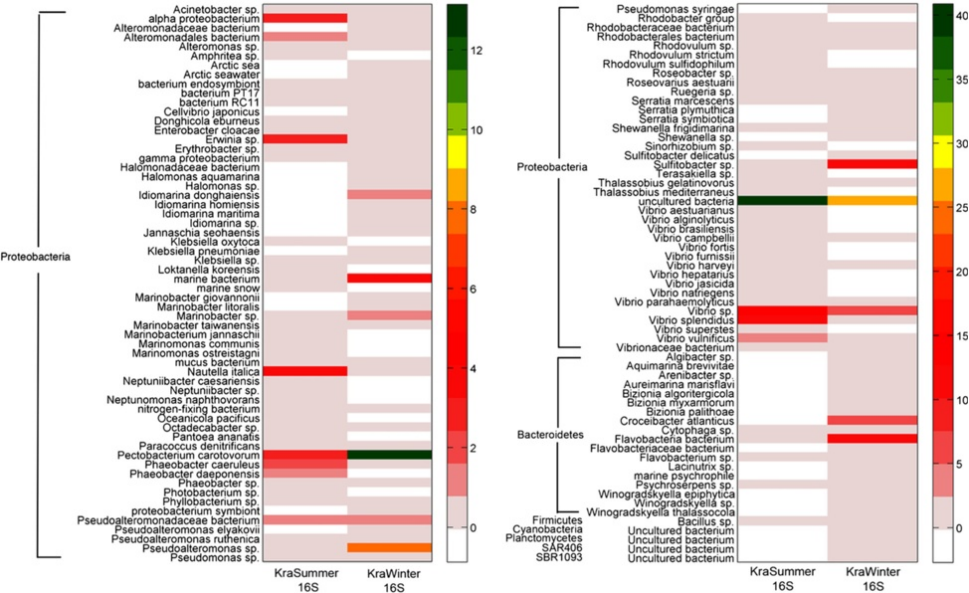
**Frequency diagram of free-living prokaryotic species in summer and winter seasons.** A percent relative abundance of each species is represented in color gradient.

Within the same phyla, the summer and winter Kra reef surface prokaryotic communities showed further characteristic species compositions as determined by low community structure relatedness Lennon (10.43%) and Yue & Clayton (8.48%) theta similarity indices. Species predominated in the summer were uncultured Proteobacteria sp., *Erwinia* sp., *Nautella italica, Vibrio* sp. and *Vibrio splendidus*; meanwhile species predominated in the winter were *Pectobacterium carotovorum, Pseudoalteromonas* sp., marine bacterium, *Sulfitobacter* sp., *Croceibacter atlanticus* and *Flavobacteria* bacterium (Figure 4). Marine bacterium represents one taxon name in Greengenes database, including isolates VS05_121 (from South Pacific), B36 (North sea of the United Kingdom) and KS-9-10-4 (South Korea), for instances.

### Eukaryotic diversity of summer and winter Kra reef surface

As an additional analysis to the mg-RAST, the 18S rRNA sequence profiles of summer and winter Kra prokaryotes were also species annotated by BLASTN [31] with E-value ≤10^−5^ against NCBI non-redundant [23], EMBL [26,27] and SILVA [28] databases and many additional unclassified (derived from Eukaryota) by mg-RAST were defined. Table 2B demonstrated the fewer eukaryotic phyla diversity in the summer than in the winter.

While fungal phyla were not abundant and thus did not demonstrate much difference, the compositional structures of the protist, plant and animal phyla were greatly different between the seasons (Table 2B). Overall, animals Brachiopoda, Mollusca and Annelida, and protists Dinophyta, constituted 87.12% of the total phyla compositions in the summer. On the other hand, plants Bacillariophyta and Stramenopiles constituted 87.30% of all phyla in the winter. Indeed, several plant phyla were missing in the summer (Table 2B).

**Figure 5:**
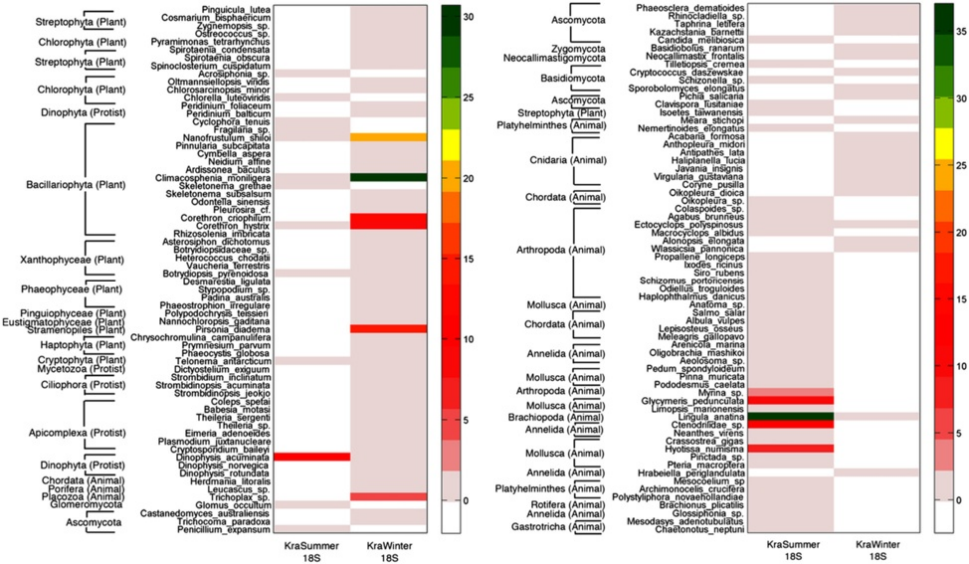
**Frequency diagram of free-living small eukaryotic species in summer and winter seasons.** Species and phyla are ordered according to their relative genetic distances among one another. A percent relative abundance of each species is represented in color gradient. In parenthesis represents a kingdom name to the phylum that belongs to the kingdom other than fungi.

Within the same phyla, the summer and winter Kra reef surface eukaryotic communities showed further characteristic species compositions as determined by low Lennon (7.81%) and Yue & Clayton (0.32%) theta similarity indices. These similarity indices were even lower than those belonged the 16S rRNA profiles. Species predominated in the summer were *Dinophysis acuminata* (phylum Dinophyta, kingdom Protist), *Myrina* sp. (Arthropoda, Animal)*, Glycymeris pedunculata* (Mollusca, Animal)*, Lingula anatina* (Brachiopoda, Animal)*, Ctenodrilidae* sp. (Annelida, Animal) and *Hyotissa numisma* (Mollusca, Animal). Species predominated in the winter were *Nanofrustulum shiloi* (Bacillariophyta, Plant)*, Climacosphenia moniligera* (Bacillariophyta, Plant)*, Corethron criophilum* (Bacillariophyta, Plant)*, Corethron hystrix* (Bacillariophyta, Plant)*, Pirsonia diadema* (Stramenophiles, Plant)*,* and *Trichoplax* sp. (Placozoa, Animal), for examples (Figure 5).

### Metabolic subsystem analysis of bacterial communities in summer and winter Kra reef surface

**Figure 6:**
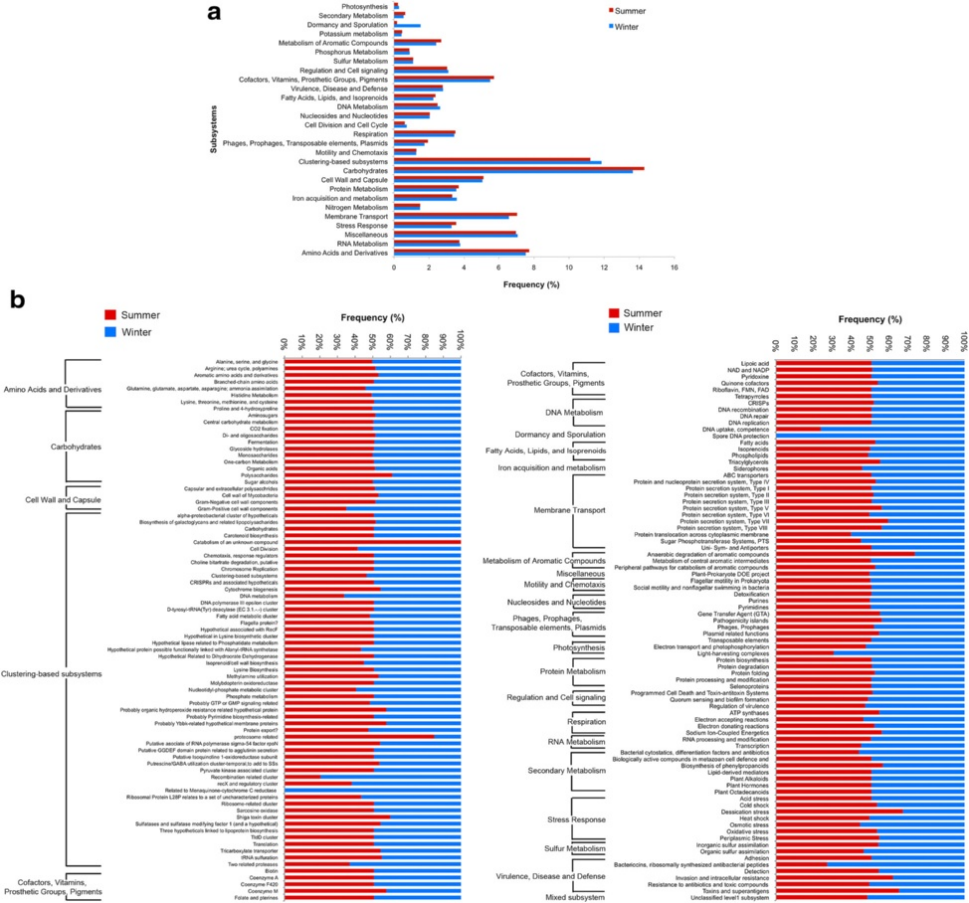
**Metabolic potentials of bacteria communities in summer and winter Kra reef surface water. a**, 28 major subsystems. **b**, further subdivisions based on functional groups in each subsystem. Subsystems and functional groups are classified by mg-RAST [29,30,52].

A total of 28 metabolic subsystems were found in summer and winter bacterial communities of Kra reef surface. Figure 6a demonstrated quite similarities in the distribution of these 28 subsystems between seasons, except in the subsystem of dormancy and sporulation that were more preponderated in the winter season. Overall, the main bacterial metabolic subsystems entailed carbohydrates, clustering-based subsystems, and amino acids and derivatives, respectively. Analysis of the distributions of functional groups within each subsystem showed that summer contained higher functional groups in: aromatic amino acids and derivatives (subsystem amino acids and derivatives); polysaccharides (carbohydrates); catabolism of an unknown compound, probably organic hydroperoxide resistance related hypothetical protein, probably Ybbk-related hypothetical membrane proteins, proteosome related, Shiga toxin cluster (clustering-based subsystems); coenzyme M (cofactors, vitamins, prosthetic groups, pigments); anaerobic degradation of aromatic compounds (metabolism of aromatic compounds); desiccation stress (stress response); invasion and intracellular resistance, toxins and superantigens (virulence, diseases and defense). For the winter the higher functional groups were: gram-positive cell wall components (cell wall and capsule); DNA metabolism, nucleotidyl-phosphate metabolic cluster, recombination related cluster, recX and regulatory cluster, related to Menaquinon-cytochrome C reductase, two related proteases (clustering-based subsystems); DNA uptake, competence (DNA metabolism); spore DNA protection (dormancy and sporulation); protein translocation across cytoplasmic membrane (membrane transport); light-harvesting complexes (photosynthesis); bacteriocins, ribosomally synthesized antibacterial peptides (virulence, diseases and defense) (Figure 6b).

### Global comparative analyses of prokaryotic profiles representing summer and winter Kra reef surface against coastal Tha Wang and Tham Phang of Sichang island, 73 GOS sites, and 4 Northern Line islands sites

**Figure 7:**
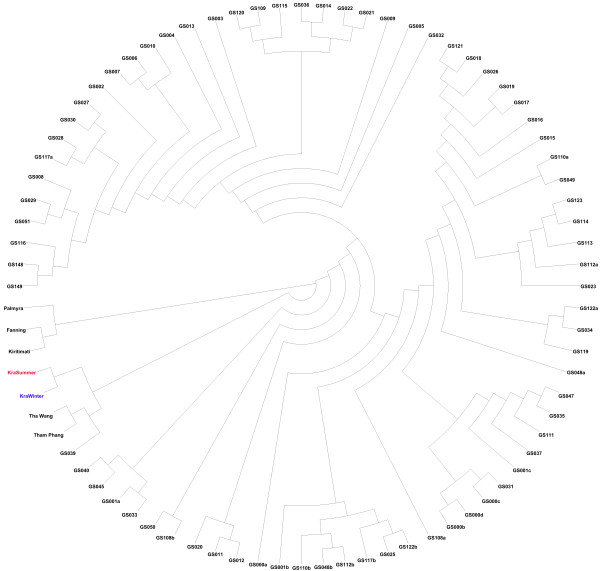
**UPGMA tree showing distances of prokaryotic communities between Kra island reef surface, Sichang coast, 73 GOS sites and 4 Northern Line Islands sites.** Distances between pairs of communities were based on similarity coefficients Thetayc computed by mothur [35].

**Figure 8:**
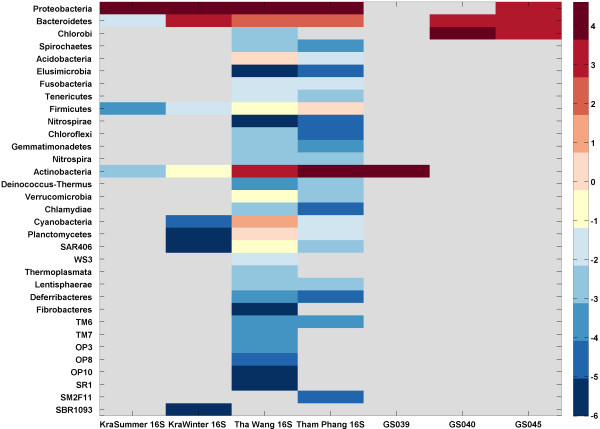
**Frequency diagram comparing prokaryotic phyla compositions representing summer and winter Kra reef surface, Tha Wang and Tham Phang coasts of Sichang island, GS039, GS040 and GS045.** Diagram was displayed in gradient colors. Phyla with red color convey that they were higher-represented when comparing with the average phyla compositions. Phyla with blue color mean that they were under-represented when comparing with the average phyla compositions. Grey color means no species present at that phylum.

Similarity between pairs of prokaryotic community structures were determined by theta similarity coefficients Yue & Clayton (Thetayc) and Smith (Thetan), using mothur [34,35]. These similarity coefficients were used for principle coordinate analysis (PCoA) and un-weighted pair group method with arithmetic mean clustering (UPGMA). Results indicated the prokaryotic communities between the summer and winter Kra reef surface were most closely related, followed by coastal Tha Wang and Tham Phang of Sichang island, GS039 (S3.343333, W101.373889, sample depth at 2 m, water depth at >4000 m, Open Ocean site, Tropical South Pacific region from INTERNATIONAL), GS040 (S4.498889, W105.070000, sample depth at 2.2 m, water depth at >4000 m, Open Ocean site, Tropical South Pacific region from INTERNATIONAL), GS045 (S9.017500, W127.767222, sample depth at 1.7 m, water depth at >4000 m, Open Ocean site, Tropical South Pacific region from INTERNATIONAL, location of 400 miles from F. Polynesia), respectively (Figure 7). Specifically, the similarity coefficients revealed that the winter prokaryotic community of the Kra reef surface was more closely related to those of Tha Wang (Thetayc summer = 0.963739, Thetayc winter = 0.987038) and Tham Phang (summer = 0.973696, winter = 0.98611) than the summer. In contrast, the summer rather than the winter Kra reef surface prokaryotic community was more closely related to those of GS039 (summer = 1.000000, winter = 0.998852), GS040 (summer = 1.000000, winter = 0.999934) and GS045 (summer = 1.000000, winter = 0.99961).Figure 8 compared the prokaryotic phyla distributions among Kra reef surface, Sichang island coast and the three related GOS communities. The relatedness between the winter Kra and the coastal Sichang prokaryotic communities was associated by the great presentation of Proteobacteria and Bacteroidetes, and the shared presence of Cyanobacteria, Planctomycetes, SAR406 and SBR1093. The relatedness between the summer Kra and GS039, GS040 and GS045 prokaryotic communities involved the rather restraint phyla distribution of simply Proteobacteria and Bacteroidetes (Figure 8). Figure 8 also revealed the more diversified prokaryotic phyla in Thai marine habitats: Tha Wang followed by Tham Phang, winter Kra reef surface, summer Kra reef surface, GS045, GS040 and GS039, respectively.

## Discussion

To unravel the microbial biodiversity in the tropical reef surface of Kra island, a home of fruitful corals, and surrounding animals and plants, this present study was conducted to obtain the prokaryotic and eukaryotic databases for the summer and winter seasons of this coral reef surface. Free-living microorganisms and particles of approximately 0.22-30 μm in diameter were captured, their metagenomes were extracted, and high number of reliable reads (97.43%) and respectable read length (186 nt) were retrieved.

Total 16S and 18S rRNA gene analyses showed that bacteria the major domain of lives in this marine habitat (Figure 2), supporting the fact that bacteria are ubiquitous on almost every environment [2,4]. Meanwhile, archaea was not found in this marine habitat, consistent with current GOS analysis and previous literatures that reported 0 to <1.0% archaea in typical marine microbial community [2,9,17]. Current GOS reported 0% archaea in estuary marine, 0.2% open-ocean, and 0.4% coastal water (average 0.3% for entire GOS) [2]. Wegley et al. [17] reported <1% archaea in coral fragments. As archaea should be more present in contaminated and polluted water environments [2,4], the absence of archaea might in part highlight the cleanliness and non-polluted Kra coral reef water, allowing bacteria to be overpopulated.

As previous studies found that not only local environmental factors like sunlight, sea depth and substrate inclination but also seasons could exert a strong influence on the structural and functional biodiversity of coral benthic communities [36], our data demonstrated season could impact different microbial population structures and functions. The differences appeared since the domain and taxon distribution patterns (Figures 2 and 3). The greater bacterial proportion in the summer (Figure 3) was perhaps associated with its less abundant eukaryotes. The less animal disturbance due to time of dormancy and sporulation in the winter might support the growth of photosynthetic lives, and thus the biodiversity of eukaryotes in the winter.

For bacteria, the study found Proteobacteria to be predominant in the summer, while not only Proteobacteria but also Bacteroidetes were prevalent in the winter (Table 2A). Changing pattern of bacterial phyla composition was consistent with recent findings by Gilbert and colleagues [9,10], stating microbial communities did change over time, between seasons, and between day and night. In addition, the greater bacterial diversity in the winter (Figure 4) was consistent with these 6-year analyses of the bacterial structures, representing the coastal Western English Channel [9] and the coastal Plymouth in UK [10], that proved the bacterial diversity was highest in winter and at night, and showed season as one important factor for microbial diversity. Note these studies used the same experimental methods as ours. Furthermore, most bacterial phyla detected in the winter Kra reef surface were also found on the coral fragments from Bocas del Toro, Panama (N9.3306 W82.2494) (Proteobacteria 68%, Firmicutes 10%, Cyanobacteria 7% and Actinobacteria 6%) [17]. Note this study was, again, conducted using the same method as ours, and the different bacterial compositions between this and ours could be attributed to different marine geography and sampling time.

The prevalence of Bacteriodetes (Table 2B) in the winter Kra reef surface was not uncommon, as this phyla was also found in various marine environments [4,37]. Species in this phyla could serve marine lives pathogens and opportunistic human pathogens, for examples, *Croceibacter atlanticus*, *Flavobacterium* sp. and *Psychroserpens* sp. (Figure 4). *C. atlanticus* in a family *Flavobacteriaceae* was isolated from Bermuda Atlantic Ocean [38]. This species and other *Flavobacterium* sp. could cause fish diseases and are opportunistic pathogens for humans due to, for instance, their chorismate biosynthesis pathway [39]. *Psychroserpens* sp. is also in a family *Flavobacteriaceae*, and was found strictly associated with some fish diseases, such as amoebic gill disease [40]. Yet, bacterial pathogens were also noticed in the summer. *Vibrio* sp. (13.42% summer, 6.94% winter) and *Pectobacterium carotovorum* (13.15% summer, 2.86% winter) could cause diseases in marine animals and plants, respectively (Figure 4) [5,41]. *Vibrio* sp. in undercooked seafood serves as a frequent cause of foodborne diseases in humans [42]. Hence, summer and winter Kra reef surface contained species that might be pathogenic to marine lives and humans.

Additionally, the findings of SAR406 and SBR1093, which are relatives of green sulfur bacteria *Chlorobium*, in the winter (Figure 4), though supported the diversity peak in the winter, might pose a threat. The reason was because *Chlorobium* could live in harsh-living conditions and could out-compete the growth of other photosynthetic microbes in restricted nutrient condition. *Chlorobium* is a photolithotrophic oxidizer of sulfur, and could also produce huge quantities of methane and hydrogen sulfide, causing global warming and acid rain [32]. In Thailand, *Chlorobium* was previously reported in a human-polluted Tha Wang coast of Sichang island [4]. Overall, the low Lennon and Yue & Clayton theta similarity indices represented the dynamic of bacterial compositions between summer and winter seasons.

In support of the aforementioned results and previous reports [9,10], the greatly diversified eukaryotic phyla and species, except for fungi, were found in the winter (Table 2B and Figure 5). Fungi were presented in a minor portion in both seasons, although their function could involve nutrient recycling like bacteria yet some could serve pathogens to the marine lives in the food webs [17]. Because several fungal species are not independent-living, the 2-step filtration method that selected for ~0.22-30 μm microorganisms or particles (extracellular DNAs) did not target these living-dependent fungi. However, the filtration method is widely practiced among metagenomic scientists [1,2,22], serving as an appropriate method for collection of microbial-size organisms and particles in the seawater.

Plant species, mainly *Climacosphenia moniligera* from phylum Bacillariophyta (0.12% summer vs. 30.70% winter: Figure 5), were abundant in the winter. These unicellular diatoms were found commonly along the corals, and could function as producer to this food chain [17]. Thus, the abundant plant species might infer the more productive coral ecosystem during the winter. On the other hand, the larger proportion of animal species in the summer (Figure 5) could likely be extracellular DNA, as many detected animals were of larger size than 30 μm. A significant proportion of brachiopods *Lingula anatina* in the summer (37.07% summer, 0.01% winter; Figure 5) was questionable; perhaps marine animal activities were so high in the summer that their extracellular DNAs from larvae or any dead debris were detected [4]. The explicit differences in the 18S rRNA gene profiles between these seasons resulted in the very low Lennon and Yue & Clayton theta similarity indices.

As coral microorganisms could help the coral animals to adjust to altering niches [15-17], the metabolic potentials for the summer and winter Kra reef surface bacterial communities were revealed to better understand this coral reef surface biodiversity pattern and its species-species interactions. 28 metabolic subsystems were discovered. Subsystems of carbohydrates, clustering-based subsystems, and amino acids and derivatives, were highly presented in both seasons, as these subsystems were important for any ecosystem. Finding of the greater dormancy and sporulation rate in the winter (Figure 6a) supported the general knowledge of this season for animal dormancy and sporulation. Functional groups of membrane transport and photosynthesis that were higher in the winter (Figure 6b) suggested the enriched food chain, supporting the diversity peak. These changes in key subsystems (i.e. photosynthesis) among seasons were consistent with previous studies by Gilbert et al. [9] and our water property measurements showing the greater percent dissolved oxygen and lower suspension solids in the winter (Table 1). Further, algae could stimulate bacterial respiration, and were found greater in the winter [43]. Consequently, changes in bacterial patterns between seasons have intricate relationships with other marine lives, and affect the metabolic potentials of the ecosystems potentially in a way characteristic to individual seasons.

**Figure 9:**
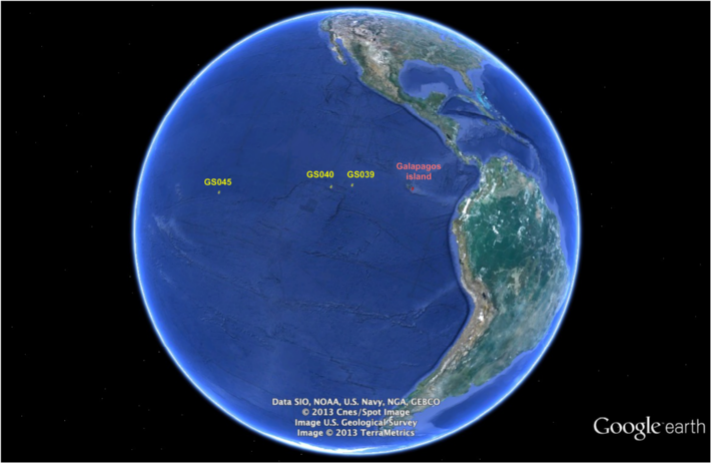
**Satellite map of GS039, GS040 and GS045.** The map was taken from Google Satellite Map, on 21 May 2013.

Finally, the prokaryotic profiles representing Kra reef surface were compared to our Thai marine datasets, coastal Tha Wang and Tham Phang of Sichang island, 73 GOS and 4 Northern Line Islands datasets. The closer prokaryotic community structures between the Kra reef surface and the Sichang island (Figure 7) highlighted community relatedness due to oceanographic position (tropical climate). The next close community structures GS039, GS040 and GS045 were also probable, since these three sites represent open ocean Tropical South Pacific at coordinates S3.343333 W101.373889 (temperature 28.6°C), S4.498889 W105.070000 (27.8°C), and S9.017500 W127.767222 (28.3°C), respectively (Figure 9) [37]. These oceanographies shared relatively warm temperature of above 25°C. The great prokaryotic diversity of Sichang islands, and also the Kra reef surface although with the less degree (Figure 8), highlighted the rich microbial biodiversity in the Thai maritime zone.

The present study provided complete independent-living prokaryotic and eukaryotic profiles of 0.22-30 μm of summer and winter Kra reef surface. Still, time series and rainy season analyses could be performed next to understand the seasonal pattern of the Kra reef surface community. Also, microbial communities of the corals and the coral sediments should be determined to gain complete understanding of the coral reef ecosystem at the Kra island.

## Conclusions

Summer and winter showed differences in species richness and evenness in Kra reef surface prokaryotic and small eukaryotic populations. The 16S and 18S rRNA gene databases serve as the baseline for future studies of coral reef microbes in Thai maritime, helping to elucidate association of microbes in differing oceanography and human impacts. Additionally, the data helped understand how Kra reef surface microbial community and metabolic potentials are associated with seasons and those of the other marine ecosystems worldwide.

## Methods

### Reef surface water sampling

Reef surface water samples (<1 meter depth) around Kra island coral reefs were collected into separated sterile containers in May 2011 and in January 2012, between 11:00–14:00 hrs. Kra island is very small and comprises three small islets (Kra Yai, Kra Klang and Kra Lek), so simultaneously three independent water samples were collected around these islets per time period to represent the site. The sampling positions were at N8.40116 E100.73232, N8.39768 E100.73643 and N8.36135 E100.73524, and more than replicate samples were collected per sample position, per time. Sample collection site and positions were selected with support from Marine and Coastal Resources Center, Lower Gulf of Thailand. This research area did not require any permission or ethical approval to work on the coral reef. General water characteristics measured on-site included pH, salinity (parts per thousand: ppt), temperature, dissolved oxygen and suspension solids. All samples were transported on ice, stored in 4°C and were processed for the next steps within 14 days.

### Metagenomic extraction and DNA quality examination

The metagenomic DNA extraction was performed separately for each of the three oceanographic positions representative to the Kra island. Each water sample was poured through four-layered sterile cheesecloth to remove debris and large-size organisms of >30 μm [4]. Then, independent-living prokaryotes and eukaryotes and particles of sizes ≥0.2 μm were captured using a sterile 0.22-μm filter (Merck Millipore, Massachusetts, USA). Total nucleic acids were extracted, and appeared around 40 kb in size, according to Metagenomic DNA Isolation Kit for Water (Epicentre, Wisconsin, USA) [22]. The extracted metagenomes were analyzed for quality and concentration by agarose gel electrophoresis and A_260_/A_280_ nanodrop spectrophotometry.

### Pre-tagged 16S and 18S rRNA sequence libraries preparation and pyrosequeing

The 16S and 18S rRNA gene library constructions were performed separately for each of the three oceanographic positions representative to the Kra island. For broad-range amplification of prokaryotic 16S rRNA and 18S rRNA genes, universal prokaryotic 338 F and 803R primers [4,44-46] and universal eukaryotic 1A and 516R primers [4,47-49] were used. For sample labelling, 8-nt pyrotag sequences were added to each primers [50]. The primer sequences were listed in Additional file 3: Table S2. For each sample, a 50-μl PCR reaction comprised 1× EmeraldAmp® GT PCR Master Mix (TaKaRa, Shiga, Japan), 0.3 μM of each primer, and 100 ng of the metagenome. The PCR conditions were 95°C for 4 min, and 30–35 cycles of 94°C for 45 s, 50°C for 55 s and 72°C for 1 min 30 s, followed by 72°C for 10 min. PCR products of about 466 (16S rRNA) and 560 (18S rRNA) nucleotides in length were excised from agarose gels, and were purified using PureLink® Quick Gel Extraction Kit (Invitrogen, New York, USA). To minimize stochastic PCR biases, two PCRs were performed per sample, and three samples were per season, yielding 6 PCR products to be pooled for pyrosequencing per sample period. 175 ng each of the pyrotagged summer and winter 16S rRNA gene amplicons and 50 ng each of the pyrotagged summer and winter 18S rRNA gene amplicons were pyrosequenced on an eight-lane Roche picotiter plate. In brief, the 454-sequencing adaptors were ligated to all 16S and 18S rRNA fragments. The reaction was purified by MinElute PCR Purification Kit (Qiagen), and pyrosequencing was performed using the 454 GS FLX system (Roche, Branford, CT) at the in-house facility of the National Center for Genetic Engineering and Biotechnology, according to the recommendations of the supplier.

### Sequence annotation and microbial composition analyses

Sequences were categorized based on their appended pyrotag-sequences, and sequences of less than 50 nucleotides were removed. Sequences corresponding to summer and winter seasons were overviewed for domain and taxonomic abundances using mg-RAST [29,30] with default parameters. Species were annotated by BLASTN [31] with E-value ≤10^−5^, against 16S rRNA gene databases including NCBI non-redundant [23], RDP [24] and Greengenes [25], and for 18S rRNA genes the databases included NCBI non-redundant [23], EMBL [26,27] and SILVA [28]. Evolutionary distances and phylogenetic tree were computed with default thresholds (E-value ≤ 10^−8^, similarity score ≥ 80%). Species (or phylum) percent relative abundance is the frequency of reads in the species (or phylum) divided by the total number of the identified reads. Similarity indices of taxonomic compositions between comparing communities were determined using Lennon and Yue & Clayton similarity indices in mothur [31,51]. The data were also compared with those of the 73 GOS (https://portal.camera.calit2.net/gridsphere/gridsphere) [1,3] and 4 sites of Northern Line Islands (Fanning, Kiritimati, Palmyra and Kingman) database [5], using Yue & Clayton theta similarity coefficients (Thetayc) and Smith theta similarity coefficient (Thetan) in mothur [34,35]. The closer the similarity coefficient to 0.000 indicated the more similarity in community structures. PCoA was plotted in three-dimensions using mothur [35]. All 16S rRNA nucleotide sequences were aligned, and an un-weighted pair group method with arithmetic mean (UPGMA) clustering were constructed at distance of 0.20 using mothur [35]. The results were manually inspected to ensure properly sequence annotation, clustering, and phylogenetic tree relationship. Further, varied sequencing depths were analyzed by (i) random selections of equal number of reads between summer and winter, and examination of their taxonomic and phyla distribution patterns against those constructed by the entire datasets; and (ii) rarefaction curve of number of phyla on Y-axis against number of random reads on X-axis.

### Bioinformatics for functional subsystem analyses

Each bacterial species comprises own sets of metabolic and functional group potentials, and these information were available from mg-RAST server [30,52]. The 16S rRNA gene profiles were thereby characterized into potential metabolic subsystems and functional groups based on their BLASTN species annotation [29].

### Availability of supporting data

All nucleic acid sequences in this study were deposited in an open access repository named Sequence Read Archive (SRA) database of NCBI, accession number SRP041071 (http://trace.ncbi.nlm.nih.gov/Traces/sra/).

## Competing interests

The authors declare that they have no competing interests.

## Authors’ contributions

NS conceived of the study, performed molecular biology experiments, participated in and coordinated the data analysis, drafted and revised the manuscript. AW performed data analysis. AA participated in study design. SM collected samples. SCT and DS carried out pyrosequencing. SDT participated in data analysis, and helped draft the manuscript. All authors read and approved the final manuscript.

## Supplementary Material

Additional file 1: Figure S1 16S and 18S rRNA genes overview for taxonomic compositions of normalized sequencing depth. Each season data comprised 10,000 and 1,600 random reads of 16S and 18S rRNA sequences, respectively. Taxons with minor abundance were shown in corresponding colors but were not presented in the circular diagram. Click here for file

Additional file 2: Table S1 Percent compositions of prokaryotic **(A)** and eukaryotic **(B)** phyla after pyrosequencing depths were normalized. Each identified read was classified in its corresponding phylum. The proportional percentage of each phylum was calculated by dividing the number of the identified reads in a phylum with the total number of the identified reads. Click here for file

Additional file 3: Table S2 Pyrotagged 16S and 18S rRNA gene primers. Italic sequence denotes 8-nt pyrotag sequence. Click here for file
