# Supplementary figures and images for: Structural and functional diversity of free-living microorganisms in reef surface, Kra island, Thailand
Source: BMC Genomics. 2014 Jul 18;15:607. doi: 10.1186/1471-2164-15-607 (PMC4223561; doi:10.1186/1471-2164-15-607)

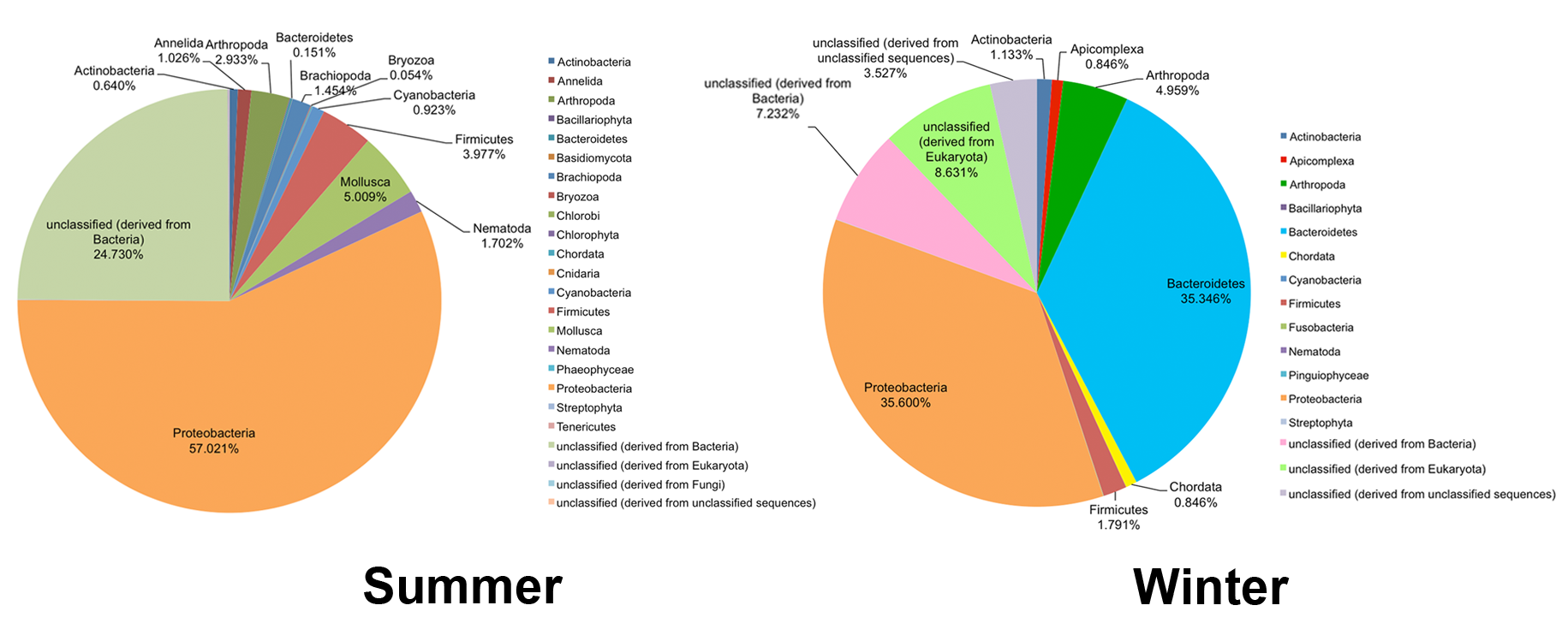

Supplement: Additional file 1: Figure S1 — 16S and 18S rRNA genes overview for taxonomic compositions of normalized sequencing depth. Each season data comprised 10,000 and 1,600 random reads of 16S and 18S rRNA sequences, respectively. Taxons with minor abundance were shown in corresponding colors but were not presented in the circular diagram. [file 1471-2164-15-607-S1.tif]
