# Supplementary material for: Structural and functional diversity of free-living microorganisms in reef surface, Kra island, Thailand
Source: BMC Genomics. 2014 Jul 18;15:607. doi: 10.1186/1471-2164-15-607 (PMC4223561; doi:10.1186/1471-2164-15-607)
Supplement: Additional file 2: Table S1 — Percent compositions of prokaryotic (A) and eukaryotic (B) phyla after pyrosequencing depths were normalized. Each identified read was classified in its corresponding phylum. The proportional percentage of each phylum was calculated by dividing the number of the identified reads in a phylum with the total number of the identified reads. [file 1471-2164-15-607-S2.doc]

# Additional file 2: Table S1

## Supplemental table 1 - Percent compositions of prokaryotic (A) and eukaryotic (B) phyla after pyrosequencing depths were normalized

Each identified read was classified in its corresponding phylum. The proportional percentage of each phylum was calculated by dividing the number of the identified reads in a phylum with the total number of the identified reads.

**A.**

| **Phyla** | **Summer (%)** | **Winter (%)** |
| --- | --- | --- |
| Proteobacteria | 99.609 | 63.717 |
| Actinobacteria | 0.103 | 0.597 |
| Bacteroidetes | 0.288 | 35.646 |
| SAR406 | - | 0.010 |
| Planctomycetes | - | 0.010 |
| Cyanobacteria | - | 0.020 |

B.

| **Phyla (Kingdom)** | **Summer (%)** | **Winter (%)** |
| --- | --- | --- |
| Ascomycota (Fungi) | 0.945 | 1.892 |
| Basidiomycota (Fungi) | 0.236 | - |
| Glomeromycota (Fungi) | 0.118 | - |
| Neocallimastigomycota (Fungi) | - | 0.065 |
| Apicomplexa (Protist) | - | 1.370 |
| Ciliophora (Protist) | - | 1.305 |
| Dinophyta (Protist) | 10.744 | 0.391 |
| Mycetozoa (Protist) | - | 0.326 |
| Bacillariophyta (Plant) | 1.653 | 69.733 |
| Chlorophyta (Plant) | 0.236 | 0.261 |
| Cryptophyta (Plant) | 0.354 | 0.261 |
| Haptophyta (Plant) | - | 0.065 |
| Phaeophyceae (Plant) | - | 0.391 |
| Pinguiophyceae (Plant) | - | 0.261 |
| Stramenopiles (Plant) | - | 16.112 |
| Streptophyta (Plant) | 0.118 | 0.065 |
| Xanthophyceae (Plant) | 0.118 | 0.718 |
| Annelida (Animal) | 15.821 | - |
| Arthropoda (Animal) | 5.903 | 0.065 |
| Brachiopoda (Animal) | 37.662 | - |
| Chordata (Animal) | 0.945 | 0.913 |
| Cnidaria (Animal) | - | 0.652 |
| Gastrotricha (Animal) | 0.236 | - |
| Mollusca (Animal) | 23.259 | - |
| Placozoa (Animal) | - | 5.088 |
| Platyhelminthes (Animal) | 1.063 | - |
| Porifera (Animal) | - | 0.065 |
| Rotifera (Animal) | 0.590 | - |
