# Supplementary material for: Structural and functional diversity of free-living microorganisms in reef surface, Kra island, Thailand
Source: BMC Genomics. 2014 Jul 18;15:607. doi: 10.1186/1471-2164-15-607 (PMC4223561; doi:10.1186/1471-2164-15-607)
Supplement: Additional file 3: Table S2 — Pyrotagged 16S and 18S rRNA gene primers. Italic sequence denotes 8-nt pyrotag sequence. [file 1471-2164-15-607-S3.doc]

**Additional file 3: Table S2 - Pyrotagged 16S and 18S rRNA gene primers**

Italic sequence denotes 8-nt pyrotag sequence.

| **Samples** | **Forward sequence (5´ -> 3´)** | **Reverse sequence (5´ -> 3´)** |
| --- | --- | --- |
| Summer 16S rRNA | *TGTACGTG*ACTCCTACGGGAGGCAGCAG | *TGTACGTG*CTACCAGGGTATCTAATC |
| Summer 18S rRNA | *ACACACAC*CTGGTTGATCCTGCCAGT | *ACACACAC*ACCAGACTTGCCCTCC |
| Winter 16S rRNA | *AGACGACG*ACTCCTACGGGAGGCAGCAG | *AGACGACG*CTACCAGGGTATCTAATC |
| Winter 18S rRNA | *TGTAGACG*CTGGTTGATCCTGCCAGT | *TGTAGACG*ACCAGACTTGCCCTCC |
